# Physical and Mental Disabilities among the Gender-Diverse Population Using the Behavioral Risk Factor Surveillance System, BRFSS (2017–2019): A Propensity-Matched Analysis

**DOI:** 10.3390/healthcare9101285

**Published:** 2021-09-28

**Authors:** Jennifer R. Pharr, Kavita Batra

**Affiliations:** 1 Department of Environmental and Occupational Health, School of Public Health, University of Nevada, Las Vegas, NV 89119, USA; 2 Office of Research, Kirk Kerkorian School of Medicine, University of Nevada, Las Vegas, NV 89102, USA; Kavita.batra@unlv.edu

**Keywords:** physical disability, mental disability, transgender, propensity score matching, Behavioral Risk Factor Surveillance System

## Abstract

This propensity-matched analysis utilized the publicly available Behavioral Risk Factor Surveillance System (2017–2019) data to compare the burden of disabilities among transgender/non-binary (TGNB) and cisgender groups. The groups were matched (1:1 ratio) on demographic variables using Nearest Neighborhood Matching. Categorical variables were compared among groups using a Chi-square analysis to test differences in the proportions. Multivariate logistic regression analysis was fit to predict the likelihood of the physical and mental disabilities among the TGNB group compared with the cisgender group while controlling for healthcare access factors, income, and employment. Survey weights were included in the model to account for the complex survey design. In a weighted sample of 664,103 respondents, only 2827 (0.4%) self-identified as TGNB. In the matched sample, a higher proportion of the TGNB group belonged to the low-income group (39.5% vs. 29.8%, *p* < 0.001), were unable to work (12.5% vs. 8.6%, *p* < 0.001), and delayed care due to cost barriers (19.0% vs. 12.4%, *p* < 0.001). Compared with the cisgender group, the odds of having difficulty making decisions were 1.94 times higher (95% CI: 1.67–2.27) and odds of difficulty walking were 1.38 times higher (95% CI: 1.19, 1.59) among the TGNB group. Additionally, the TGNB group had 59.8% higher adjusted odds ratio (aOR) (aOR 1.598, 95% Confidence interval (CI): 1.256, 2.034) of experiencing difficulty dressing and 83.3% higher odds (aOR 1.833, 95% CI: 1.533, 2.191) in having difficulty doing things alone. The findings of this study advocate for developing policies and interventions to deliver culturally competent care to the TGNB population with disabilities.

## 1. Introduction

Transgender and gender nonbinary (TGNB) persons have gender identities, expressions, or behaviors not traditionally associated with the sex they were assigned at birth [1]. In the United States (U.S.), it is estimated that TGNB persons constitute 6% of the population, or 1.4 million people [2]. Despite the growing population, visibility, and acceptance of gender minorities in the U.S., evidence continues to show wide disparities in social determinants of health (income, employment, education) and health outcomes among this population compared with cisgender (non-transgender) populations [3,4,5,6,7,8,9,10,11]. The minority stress theory posits that TGNB people are at greater risk of experiencing worse health outcomes due to stigma and its resulting discrimination [12,13,14,15]. The stigma that they experience may be at the individual, interpersonal, and societal/structural levels and occurs because their gender identity lies outside of the cisgender, binary norm [12,13,14,15]. Identity-related stigmatization results in employment discrimination, public services discrimination, macro and micro aggressions, and internalized transphobia, which leads to elevated levels of stress [12,13,14,15].

Minority stress has been linked to an increased burden of mental health disorders and chronic diseases among sexual and gender minority populations, particularly among TGNB people [12,14]. Studies reported a higher prevalence of mental health disorders such as anxiety, depression, suicidality, and substance use among TGNB persons compared with their cisgender counterparts [10,12,13,14,15,16,17]. Previous studies explained increased suicidal ideation among TGNB people through gender minority stress and interpersonal factors [16,17]. Additionally, compared with the cisgender people, TGNB people are more likely to have chronic diseases, such as hypertension, hypercholesteremia, prediabetes, stroke, cardiovascular diseases, asthma, and cancers, while also engaging in more health risk behaviors including smoking and heavy drinking [10,18]. While there is a paucity of research examining disability disparities among TGNB people, a few studies found an elevated risk of mobility, cognitive, and independent living disabilities among TGNB people compared to cisgender men, the odds of which increase with age [5,19,20,21,22,23,24].

Disability is defined by the Americans with Disabilities Act (ADA) as a “physical or mental impairment that substantially limits one or more major life activities” with major life activities including, but not limited to, self-care, manual tasks, seeing, hearing, walking, standing, lifting, speaking, learning, concentrating, and communicating [25]. Reportedly, sixty-one million American adults have a disability, equaling 25.7% of the adult population [26]. Among transgender people, 39% report having a disability [26,27]. People with disabilities have been stigmatized throughout history [3,19,27]. The stigma of disability contributes to discrimination in many facets of life such as employment, education, and life decisions; stereotypes; negative attitudes; and labeling of people with disabilities [28].

Because people with disabilities are subjected to disability stigma, this could result in TGNB people with disabilities having multiple minority stress. However, the epidemiology of these two minority stressors is relatively unknown in the TGNB population. There is evidence to suggest that sexual minority people are more likely to have disabilities compared with heterosexual people and that this intersection results in poorer health and a great need for community support [29,30,31]. However, minimal research is available for gender minorities. The little research that has been conducted used uneven samples to draw conclusions Therefore, the purpose of this study was to use a propensity-matched sample to examine disabilities disparities among TGNB people compared with cisgender people. Authors hypothesize that TGNB people experience a higher burden of physical disabilities compared to their cisgender counterparts after matching on certain demographic variables.

## 2. Materials and Methods

### 2.1. Study Design and Data Source

This cross-sectional study utilized publicly available data from the Behavioral Risk Factor Surveillance System (BRFSS) for the years 2017–2019 [32]. The BRFSS is the largest, population-based, and nationwide computer-assisted telephone interview (CATI) survey conducted by the CDC, which collects data from all 50 states and selected territories of the United States (U.S.) [32,33,34]. The target population of the BRFSS survey is non-institutionalized adults aged 18 years or older [32,33,34]. The BRFSS collects information about participants’ demographics, health-related risk behaviors, chronic health conditions, and use of preventive services in the core module of the questionnaire [32]. In 2014, the BRFSS expanded the utility of the surveillance data by including an optional module related to sexual orientation and gender identity (SOGI), which was utilized by 28 states and territories in 2017, 30 states and territories in 2018, and 31 states and territories in 2019 [33,34]. To account for the bias resulting from the selection probabilities and non-response, the BRFSS utilizes a complex sampling weighting methodology, which can be viewed at https://www.cdc.gov/brfss, accessed on 24 June 2020. This helps in generating the nationally representative samples [35]. This study was deemed excluded from the ethical review by the University of Nevada, Las Vegas Institutional Review Board, because it involves secondary data analysis of publicly available, deidentified data with no direct involvement of the human subjects.

### 2.2. Participants

The study was comprised of survey participants of at least 18 years of age and who responded “yes” to the question “Do you consider yourself to be a transgender?” [36,37,38]. Participants who self-identified as Transgender, male-to-female; Transgender, female-to-male; and gender nonconforming were included in the TGNB (yes) category. Participants who responded “no” to the above question were included in a comparative cisgender (transgender “no”) group. Respondents who responded “don’t know or unsure” or “refused to answer” were excluded from the study. This methodology was consistent with the previous studies, which utilized the BRFSS data [39,40,41].

### 2.3. Variables and Measures

Variables included in this study were sociodemographic information (e.g., birth-assigned sex, education, age, race/ethnicity, marital status, employment status and income) and healthcare accessibility information (health insurance, having personal doctor, frequency of medical check-up, and delayed check-up due to cost) [36,37,38]. We recoded some variables to construct meaningful categories. For instance, unemployed includes those who were out of the labor force (e.g., retired, homemakers, students, and unable to work) and the non-white category includes Blacks, multiracial, Asian, Native Hawaiian/Pacific Islanders, and Alaska Natives/American Indians. The main outcomes for this study were physical and mental disabilities, e.g., difficulty hearing, seeing, performing routine activities such as walking, dressing, bathing, and limited ability to make decisions. We also evaluated the number of days of poor mental and physical health in the past month (i.e., 0 days, 1–13 days, or <14 days). Because some disabilities are associated with poor health, chronic conditions, and lack of access to healthcare, we included healthcare access as a demographic characteristic in this study. Additionally, we included healthcare access as a covariate in this analysis because previous research has identified that financial barriers to healthcare are predictors of disability among SGM populations [5,24].

### 2.4. Statistical Analysis

The data from the three surveys (2017, 2018, and 2019) were pooled to increase the sample size following the methodology described by the CDC [35]. The final weight was calculated and adjusted according to the proportion of the sample size in each year. To account for the sample size differences in the cisgender and transgender groups and to minimize selection bias, we used the propensity-score-matched (PSM) analysis [10,42,43]. Cisgender (control) and TGNB (case) groups were 1:1 matched on demographic variables, including birth-assigned sex, age, race, marital status, and education. Nearest Neighborhood Matching (NNM) was performed using the matching package [43] and covariate balance was assessed using cobalt [44,45], both in R [46]. Propensity scores were first estimated using a logistic regression and the probability distribution was then compared. Covariance balance in unmatched and matched sample was compared by visually inspecting the Love plots. In addition, standardized mean differences and variance ratio were used as indicators to assess covariance balance [10]. Categorical variables were compared among groups using a Chi-square analysis to test the differences in the proportions. Multivariate logistic regression analysis was fit to predict the likelihood of the physical and mental disabilities among the TGNB group compared with the cisgender group while controlling for healthcare access factors, income, and employment. Survey weights were included in the model to account for the complex survey design using a survey package in R software. The significance level was set at 5%, and all descriptive analysis of the matched samples were conducted using SPSS version 26 (IBM Corp. Armonk, NY, USA).

## 3. Results

**Figure 1:**
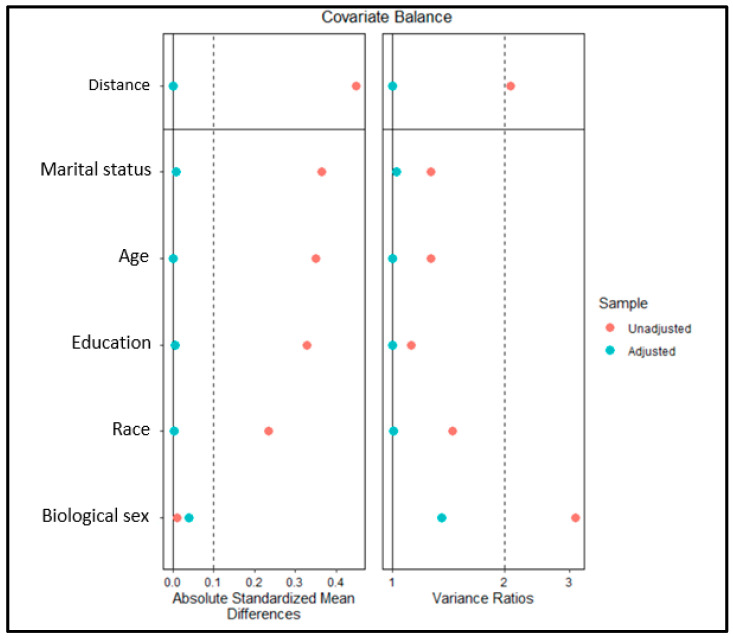
Love plot displaying covariate balance in unadjusted and adjusted samples. Legend: Absolute standardized mean differences close to zero and variance ratio close to one is indicative of a good covariate balance.

In a weighted sample of 664,103 respondents, only 2827 (0.4%) self-identified as TGNB. In an unmatched sample, a higher proportion of the TGNB group were non-white (23.2% vs. 20.5%, *p* < 0.001), Hispanic (21.0% vs. 15.8%, *p* < 0.001), aged 18–34 years (47.4% vs. 27.6%, *p* < 0.001), were never married (38.8% vs. 23.5%, *p* < 0.001), and were less educated with only a high school diploma or less (55.1% vs. 41.4%, *p* < 0.001; Table 1). The post-matched sample (*n* = 2687) had an adequate covariate balance, as indicated by absolute standardized mean differences close to zero and variance ratios close to 1, as indicated in Figure 1 and Table 2.

Upon analyzing differences in the socioeconomic, healthcare access, and cost of care barriers, significant differences among unmatched and matched samples were noted (Table 3). In a matched sample, with the exception of healthcare insurance, there were statistically significant differences in the proportion. A higher proportion of the TGNB group belong to the low-income group (39.5% vs. 29.8%, *p* < 0.001), were unable to work (12.5% vs. 8.6%, *p* < 0.001) and delayed care due to cost barriers (19.0% vs. 12.4%, *p* < 0.001; Table 3) compared with the cisgender group. However, for personal doctor and medical check-ups, a significantly higher proportion of the TGNB respondents reported having a medical checkup within the past year (76.6% vs. 70.3%, *p* < 0.001) and had at least one personal doctor (69.9% vs. 61.1%, *p* < 0.001; Table 3). The results of outcome analysis indicated that TGNB individuals were more likely to experience difficulty seeing (7.9% vs. 6.1%, *p* = 0.008) and making decisions (22.5% vs. 12.5%, *p* < 0.001, Table 4) as opposed to their cisgender counterparts. A significantly higher proportion of TGNB respondents reported difficulty walking (20.6% vs. 14.3%, *p* < 0.001), dressing (7.6 vs. 4.3%, *p* < 0.001), and doing things alone (15.4% vs. 8.3%, *p* < 0.001, Table 4). As compared with the cisgender group, the TGNB group reported having poor physical (18.4% vs. 13.9%, *p* < 0.001) and mental health outcomes (24.9% vs. 14.1%, *p* < 0.001), which persisted for at least 14 days (Table 4).

After adjusting for the healthcare access factors, cost-of-care barriers, income, and education, having difficulties making decisions, walking, dressing, and doing things alone were associated with the TGNB group. Compared with the cisgender group, the odds of having difficulties making decisions were 1.94 times higher (95% CI: 1.67–2.27) and odds of difficulties walking were 1.38 times higher (95% CI: 1.19, 1.59, Table 5) among the TGNB group. Additionally, the TGNB group had 59.8% higher odds (aOR 1.598, 95% CI: 1.256, 2.034) of experiencing difficulties dressing and 83.3% higher odds (aOR 1.833, 95% CI: 1.533, 2.191, Table 5) having difficulties doing things alone.

## 4. Discussion

Pre- and post-matching, TGNB people experience disparities in two important factors that are determinants of health (i.e., income and employment) when compared with cisgender people. This is consistent with other studies that did not use matched samples [5,6]. TGNB people tend to have lower rates of employment and make less money than their cisgender peers, possibly due to stigma at the interpersonal and structural levels. While TGNB people may have more protections under the law than their lesbian, gay, and bisexual peers due to the Title VII federal sex discrimination law, they still experience unacceptably high rates of workplace discrimination. A recent survey of TGNB people found that 44% had experienced discrimination in employment and hiring in the prior 12 months [47,48,49]. Research has also shown that sexual and gender minority adults experience discrimination in hiring practices and compensation, leading to higher unemployment and lower income despite high rates of higher education [50,51].

Access to healthcare is important for diagnosing, preventing, and treating disabilities. However, TGNB people face discrimination in healthcare that results in a multitude of barriers to accessing care, which include a lack of health insurance or insurance coverage for gender affirming care, financial barriers, transphobic healthcare providers, verbal or physical abuse and maltreatment by healthcare providers, and healthcare providers’ lack of knowledge about TGNB care [52,53,54]. Prior to matching in this sample, TGNB people in this study were less likely to report having a personal doctor or having had a medical check-up in the past year than cisgender people. However, after matching, we saw these results switch, with TGNB people being more likely to have a personal doctor (70%) or a medical checkup in the past year (77%). This is similar to results from the U.S. Transgender Survey [54]. This study demonstrates that matched results are particularly important as they provide more accurate estimates after accounting for the imbalance among covariates, which might otherwise have contributed to the hypothesized effects in the unmatched findings. Knowing the barriers to healthcare experienced by TGNB, these findings from the matched analysis are encouraging, and may be due to the healthcare needs of TGNB people, especially those who are on gender-affirming hormones or receiving care from a transgender provider.

In the matched analysis, TGNB people were more likely to report all disabilities (except difficulty hearing) and 14 days or more of poor physical and mental health. After controlling for healthcare access factors, income, and employment, TGNB people were found to have greater disability disparities including difficulties making decisions, walking, dressing, and doing things alone. Previous research using an unmatched sample also found that the TGNB people were more likely to have mobility, cognitive, and independent living disabilities compared with cisgender people, which this study confirms [5]. TGNB people in this study were almost two times more likely to have difficulties making decisions or doing things alone. This may indicate a need for help that might not be readily available for TGNB people. The largest survey of TGNB people in the U.S. found that they were more likely to be single and never married compared to the U.S. population [3]. Our findings are similar with regard to marital status. Additionally, TGNB people are more likely to experience family rejection and homelessness and are less likely to have children [3]. People with disabilities often need additional assistance with activities of daily living and usually this assistance comes from family members and friends [3,55,56]. TGNB people who have disabilities may rely on their friends for support and help rather than their family of origin. Indeed, older sexual and gender minorities are more like to rely on friends for care than do their heterosexual, cisgender peers [55,57]. Research has found that, within the sexual and gender minority community, friends who provide informal care have less access to social support, which leads to greater stress and depressive symptoms [58]. Our finding of increased risk of disability highlights the need for support services that are culturally sensitive to those with disabilities who identify as TGNB and their caregivers.

A disability may make healthcare access issues more profound for TGNB people. According to Healthy People 2020, 76.8% of adults with disabilities experience barriers that hinder their use of available healthcare and wellness services [59]. These barriers include structural barriers (e.g., lack of accessible medical equipment), financial barriers (e.g., lack of health insurance, medical equipment that is too expensive), and personal/cultural barriers (e.g., misconceptions about people with disabilities, maltreatment from healthcare providers) [19,60,61]. Healthcare barriers persist despite having two federal laws (Americans with Disabilities Act and section 504 of the Rehabilitation Act), which require healthcare providers to grant their patients with disabilities full and equal access to healthcare services and facilities [62,63,64]. Having a disability and identifying as TGNB may present a double barrier to healthcare. As mentioned above, TGNB people experience unique barriers to healthcare compared with the cisgender population. Previous research has found that TGNB people with disabilities are two times more likely to experience financial barriers to health care [57]. Additionally, financial barriers to health care, lifetime victimization, internalized stigma, and lack of social support are predictors for disability among older sexual minority adults [57]. Policies at the federal, state, and institutional levels are not only needed to address structural stigma, but need to be implemented and enforced to address healthcare barriers experienced by TGNB people and people with disabilities.

TGNB people with disabilities are likely to experience multiple minority stress due to their multiple minority identities, that of being TGNB and that of having a disability. Both groups are at an increased risk of discrimination [65]. For example, stigma and incorrect stereotypes lead some healthcare providers to see people with a disability as asexual, and, in turn, they do not offer certain services such as sexually transmitted disease testing [58,59]. On the other hand, healthcare providers may have limited knowledge of gender-affirming care. Together, these two situations would impede healthcare for a TGNB person with a disability. Individually, TGNB stigma and the stigma of disability impact depression, psychological distress, quality of life, and quality of healthcare [49]. Together, these two forms of stigma may have a profound impact on the health, well-being, and healthcare of TGNB people with disabilities. However, because of the paucity of research at the intersection of disability and TGNB identity, little is known about the health burden of this form of multiple minority stress. More research is needed at this intersection to understand the health impacts.

### Strengths and Limitations

To our knowledge, this is the first study that utilized propensity-matched analysis to investigate the burden of disabilities among TGNB people. Transgender and cisgender participants were appropriately matched on demographic characteristics to assess the true differences across groups. Like other studies, this study is not without limitations. First, the cross-sectional design of this study does not allow us to infer causality. Moreover, BRFSS utilizes a repeated cross-sectional design; it is likely that some participants were sampled across the years, which might have introduced some bias in the estimates. However, authors expect minimal effect as estimates were generated on a matched subset. Second, due to inconsistency in the method of collecting sexual orientation information, matching did not account for the sexual orientation. Third, this study may be subject to information bias, including reporting bias and recall bias. Lastly, although groups of transgender and cisgender participants were matched on demographic characteristics, a residual confounding may still exist due to differences within the subgroups.

## 5. Conclusions

TGNB people were more likely to report disabilities and poorer mental and physical health than cisgender people. The minority stress theory would posit that these disparities are, at least in part, due to the excess stigma that TGNB people experience at the structural/social, interpersonal, and individual levels. TGNB people with disabilities may experience multiple minority stress and experience both the stigma of disability and of being a gender minority. These stigma may impact other health outcomes as well as access to healthcare. However, more research is needed to examine the intersection of disability and gender minority status. Additionally, interventions (culturally competent care) and enforceable policies are needed to address the unique barriers to healthcare experienced by people with disabilities and TGNB people.

## Figures and Tables

**Table 1:** Demographic characteristics of the unmatched study population (*N* = 664,103).

Characteristics	Gender Identities		Total	*p* Value
	TGNB	Cisgender		
	*n* (weighted %)	*n* (weighted %)	*n* (weighted %)	
All	2827 (0.5)	661,276 (99.5)	664,103	-
Sex assigned at birth				
Male	1456 (51.9)	294,479 (47.7)	295,935 (48.0)	<0.001
Female	1324 (48.1)	366,243 (51.7)	36,7567 (52.0)	
Education status				
High school graduate or less	1343 (55.1)	223,742 (41.4)	225,085 (41.4)	<0.001
Attended college	723 (28.9)	183,259 (31.3)	183,982 (31.3)	
College graduate	742 (16.0)	252,304 (27.4)	253,052 (27.3)	
Age (in years)				
18–34	847 (47.4)	99,009 (27.6)	99,856 (27.7)	<0.001
35–54	700 (25.1)	172,366 (32.3)	173,066 (32.2)	
55–74	936 (20.8)	279,048 (31.0)	279,984 (30.9)	
75+	307 (6.7)	101,102 (9.2)	101,409 (9.2)	
Race/ethnicity				
White	1784 (55.8)	500,935 (63.7)	502,719 (63.7)	<0.001
Non-white	680 (23.2)	109,607 (20.5)	110,287 (20.5)	
Hispanic	337 (21.0)	46,450 (15.8)	46,787 (15.9)	
Marital status				
Married	1032 (34.3)	338,983 (51.1)	340,015 (51.0)	<0.001
Divorced	382 (9.8)	92,057 (10.9)	92,439 (10.9)	
Widowed	285 (6.2)	82,717 (7.4)	83,002 (7.3)	
Separated	80 (3.0)	14,374 (2.6)	14,454 (2.6)	
Never married	850 (38.8)	107,597 (23.5)	108,447 (23.6)	
A member of unmarried couple	181 (7.8)	21,651 (4.6)	21,832 (4.7)	

TGNB = transgender, non-binary. Unemployed includes those being out of the labor force (e.g., retired, homemakers, students, and unable to work). Non-white includes Blacks, multiracial, Asian, NH/PI, AI/AN.

**Table 2:** Summary of covariate balance for matched cohort units (*N* = 2687).

	Means Treated (Trans)	Mean Control (Cis)	SMD	VR	Standard Pair Distance
Age	2.949	2.951	−0.0015	1.001	0.0098
Biological sex	1.569	1.533	0.0386	1.353	0.0507
Race	1.843	1.849	−0.0041	0.992	0.0102
Marital status	2.988	3.004	−0.0083	0.973	0.0146
Education level	2.635	2.630	0.0047	0.998	0.0069

SMD = Standardized Mean Difference; VR = Variance ratio. Matching variables = Age, Biological sex, Race, Marital status, Education level.

**Table 3:** Socioeconomic, health-specific and cost-of-care barriers among unmatched and matched samples.

Unmatched				Matched		
Outcome	TGNB *N* (Weighted %)	Cisgender *N* (Weighted%)	*p* Value	TGNB *N* (%)	Cisgender *N* (%)	*p* Value
Income in dollars						
Up to 25 K	926 (43.7)	138,849 (26.3)	<0.001	884 (39.5)	655 (29.8)	<0.001
25–50 K	573 (19.7)	13,4271 (23.2)		549 (24.6)	533(24.2)	
50–75 K	311 (12.5)	89,208 (15.1)		304 (13.6)	338 (15.4)	
75+ K	515 (24.1)	192,787 (35.4)		499 (22.3)	675 (30.7)	
Employment status						
In labor force or working	1350 (49.2)	326,136 (56.8)	<0.001	1301 (48.4)	1385 (51.5)	<0.001
Out of labor force or unemployed	1091 (36.9)	280,908 (36.0)		1049 (39.1)	1072 (39.9)	
Unable to work	353 (13.9)	48,893 (7.2)		337 (12.5)	230 (8.6)	
Healthcare insurance						
Yes	2434 (82.2)	605,968 (87.2)	<0.001	2317 (86.7)	2332 (87.4)	0.5
No	389 (17.8)	54,374 (12.8)		354 (13.3)	337 (12.6)	
Personal doctor						
Yes, only one	1967 (67.3)	500,447 (70.6)	<0.001	1876 (69.9)	1638 (61.1)	<0.001
Yes, more than one	237 (7.4)	54,825 (7.6)		224 (8.3)	240 (8.9)	
No	619 (25.3)	10,469 (21.8)		584 (21.8)	805 (30.0)	
Delayed care due to cost						
Yes	547 (21.9)	67,990 (13.3)	<0.001	511 (19.0)	334 (12.4)	<0.001
No	2277 (78.1)	592,187 (86.7)		2173 (81.0)	2352 (87.6)	
Medical check up						
Yes, within past year	2161 (72.6)	521,770 (74.6)	<0.001	2057 (76.6)	1889 (70.3)	<0.001
Yes, within past 2 years	259 (11.6)	62,672 (11.0)		244 (9.1)	319 (11.9)	
Within past 5 years	171 (6.1)	33,406 (6.5)		163 (6.1)	204 (7.6)	
5 or more years	176 (7.9)	31,738 (5.8)		168 (6.3)	208 (7.7)	
No	57 (1.8)	10,423 (2.0)		52 (1.9)	67 (2.5)	

TGNB = transgender, non-binary.

**Table 4:** Burden of physical and mental disabilities among unmatched and matched samples.

Unmatched				Matched		
Outcome	TGNB *N* (%)	Cisgender *N* (%)	*p* Value	TGNB *N* (%)	Cisgender *N* (%)	*p* Value
Difficulty hearing						
Yes	302 (9.8)	58,784 (6.6)	<0.001	286 (10.7)	247 (9.3)	0.07
No	2493 (90.2)	596,657 (93.4)		2375 (89.3)	2411 (90.7)	
Difficulty seeing						
Yes	219 (7.8)	34,750 (5.1)	<0.001	211 (7.9)	161 (6.1)	0.008
No	2572 (92.2)	620,253 (94.9)		2448 (92.1)	2492 (93.9)	
Difficulty making decisions						
Yes	629 (27.3)	69,714 (11.4)	<0.001	598 (22.5)	323 (12.2)	<0.001
No	2158 (72.7)	584,543 (88.6)		2058 (77.5)	2328 (87.8)	
Difficulty walking						
Yes	576 (17.4)	115,267 (14.5)	<0.001	546 (20.6)	379 (14.3)	<0.001
No	2207 (82.6)	538,729 (85.5)		2108 (79.4)	2270 (85.7)	
Difficulty dressing						
Yes	217 (9.6)	29,252 (4.1)	<0.001	202 (7.6)	114 (4.3)	<0.001
No	2567 (90.4)	624,558 (95.6)		2452 (92.4)	2531 (95.7)	
Difficulty doing things alone						
Yes	427 (20.0)	50,627 (7.2)	<0.001	409 (15.4)	218 (8.3)	<0.001
No	2352 (80.0)	602,566 (92.8)		2240 (84.6)	2423 (91.7)	
General health						
Excellent	106,025 (17.2)	399 (16.9)	<0.001	380 (14.2)	388 (14.5)	<0.001
Very Good	216,242 (31.4)	787 (25.7)		754 (28.1)	825 (30.8)	
Good	209,887 (32.4)	891 (30.3)		844 (31.5)	896 (33.4)	
Fair	509 (17.6)	91,996 (14.1)		490 (18.3)	426 (15.9)	
Poor	232 (9.4)	35,467 (4.9)		213 (7.9)	146 (5.4)	
Poor physical health						
0 days	1411 (50.2)	402,618 (63.6)	<0.001	1345 (51.8)	1523 (58.0)	<0.001
1–13 days	812 (32.4)	152,847 (23.8)		772 (29.7)	738 (28.1)	
≤14 days	505 (17.4)	91,189 (12.6)		478 (18.4)	365 (13.9)	
Poor mental health						
0 days	1408 (44.9)	433,007 (64.0)	<0.001	1351 (51.5)	1577 (59.8)	<0.001
1–13 days	651 (25.0)	141,039 (23.3)		617 (23.5)	687 (26.1)	
≤14 days	694 (30.1)	75,359 (12.7)		654 (24.9)	373 (14.1)	

TGNB = transgender, non-binary.

**Table 5:** Adjusted and unadjusted odds ratio for disabilities among matched samples.

Variable	Unadjusted Odds Ratio	95% CI		*p* Value	AOR	95% CI		*p* Value
		LCL	UCL			LCL	UCL	
Difficulty hearing								
TGNB	1.175	0.982	1.406	0.07	1.066	0.888	1.280	0.5
Cisgender	REF	-	-	-	-	-	-	-
Difficulty seeing								
TGNB	1.334	1.079	1.650	0.008	1.236	0.995	1.534	0.05 *
Cisgender	REF	-	-	-	-	-	-	-
Difficulty making decisions								
TGNB	2.094	1.807	2.428	<0.001	1.948	1.673	2.269	<0.001
Cisgender	REF	-	-	-	-	-	-	-
Difficulty walking								
TGNB	1.551	1.344	1.791	<0.001	1.381	1.192	1.599	<0.001
Cisgender	REF	-	-	-	-	-	-	-
Difficulty dressing								
TGNB	1.829	1.444	2.316	<0.001	1.598	1.256	2.034	<0.001
Cisgender	REF	-	-	-	-	-	-	-
Difficulty doing things alone								
TGNB	2.029	1.705	2.415	<0.001	1.833	1.533	2.191	<0.001
Cisgender	REF	-	-	-	-	-	-	-

TGNB = transgender, non-binary. *p* values less than 0.05 are statistically significant; Adjusted odds ratios (AOR) were obtained after controlling for healthcare access factors, income, and employment; LCL-Lower Confidence Limit; UCL-Upper Confidence Limit. * Marginally Significant.

## Data Availability

Data are available in a publicly accessible repository that does not issue DOIs. Publicly available datasets were analyzed in this study. These data can be found here: https://www.cdc.gov/brfss/data_documentation/index.htm, accessed on 24 June 2020.
